# Foliar application of enriched banana pseudostem sap influences the nutrient uptake, yield, and quality of sweet corn grown in an acidic soil

**DOI:** 10.1371/journal.pone.0285954

**Published:** 2023-08-29

**Authors:** Mahammad Shariful Islam, Susilawati Kasim, Adibah Mohd Amin, Md. Khairul Alam, Mst. Fatima Khatun, Sharif Ahmed, Ahmed Gaber, Akbar Hossain

**Affiliations:** 1 Department of Land Management, Faculty of Agriculture, Universiti Putra Malaysia, Serdang, Malaysia; 2 Bangladesh Agricultural Research Institute, Gazipur, Bangladesh; 3 Centre for Sustainable Farming Systems, Future Food Institute, Murdoch University, Perth, Western Australia, Australia; 4 CSIRO Land and Water, Western Australia, Australia; 5 International Rice Research Institute, Bangladesh Office, Dhaka, Bangladesh; 6 Department of Biology, College of Science, Taif University, Taif, Saudi Arabia; 7 Division of Soil Science, Bangladesh Wheat and Maize Research Institute, Dinajpur, Bangladesh; Nuclear Science and Technology Research Institute, ISLAMIC REPUBLIC OF IRAN

## Abstract

Foliar fertilization is a reliable technique for correcting a nutrient deficiency in plants caused by inadequate nutrient supply to the roots in acid soil. Soluble nutrients in banana pseudostem sap might be effective to supplement chemical fertilizers. However, the limited nutrients in sole banana pseudostem sap as foliar fertilization may not meet-up the nutritional demand of the crop. Field trials were, therefore, conducted with the combination of soil-applied fertilizers with foliar spray of banana pseudostem sap to increase nutrient uptake, yield, and quality of sweet corn planted in acidic soil. Three treatments viz., 100% recommended dose of fertilizers (RD) as control (T_1_), 75% of RD applied in soil with foliar application of non-enriched banana pseudostem sap (T_2_), and 50% RD applied in soil with foliar spray of enriched banana pseudostem sap (T_3_) were replicated four times. The combination of soil-applied fertilizer with foliar spray of enriched banana pseudostem sap (T_3_) showed a significant increase in leaf area index (11.3%), photosynthesis (12%), fresh cob yield (39%), and biomass of corn (29%) over control. Besides, the 50% RD of soil fertilization with foliar spray of enriched pseudostem sap increased nutrient uptake in addition to an increase in sugar content, phenolic content, soluble protein, and amino acids of corn. Considering the economic analysis, the highest net income, BCR (3.74) and MBCR (1.25) values confirmed the economic viability of T_3_ treatment over the T_1_. The results suggest that foliar spray of enriched banana pseudostem sap can be used as a supplementary source of nutrients to enhance nutrient uptake by corn while increasing yield and minimizing chemical fertilizer use in acid soil.

## 1. Introduction

Sweet corn is one of the most popular cereal crops in the world [1]. The demand for sweet corn has been expanding in food commodities because of its sweetness and high supplement content [2]. It is mainly utilized for either preparation for nourishment or non-processed food products for the substitute rice. Corn production is remarkably low (average 5.2 t ha^-1^ and the total area is around 10000 ha) in Malaysia due to soil acidity [3]. Soil-applied fertilizers react with Fe, Al, and Ca-containing compounds in soils which reduces the potency of fertilizers for plants. [4, 5]. Besides, plant roots become injured and are inhibited nutrient acquisition by the roots in low-pH soil [6, 7]. Thus, a lavish dosage of chemical fertilizers is required to optimize production, which increases acidity. In this case, foliar spray can be the alternative option to feed the crops.

On the other hand, banana waste is considered an influential resource to use as organic liquid fertilizer [8]. But the producers, simply let banana pseudostem as waste after harvesting the banana [9]. Recently, the foliar application of organic liquid fertilizer has been started in different areas or on crops under organic farming practices considering the soil and plant interaction issues [10]. It is thought extremely secure and sharply responsive to nutrients than soil-applied fertilizer [11]. The negative effects of soil acidity oppose nutrient availability through the roots. The effectiveness of foliar fertilization in these circumstances is higher than that of the use of soil fertilizer. According to Kuepper [12], foliar fertilization can be 8–20 times more effective for nutrient absorption than soil application of fertilizers. This may be due to the leaves proximity, stomatal activity, and quick absorption of available nutrients from the spray solution [13]. Thus, foliar feeding of plants is expected a bypass option to minimize the uncertainty of nutrient uptake from the acidic soil.

Although banana plant especially in pseudostem contains 80–85% fluid [14] and have the opportunity to be considered an effective organic fertilizer [8, 15] due to higher concentration of NO_3_-N, NH_4_-N, K, P, Ca, Mg, Zn, and Fe in it [16–18]. Moreover, a few researchers have reported that phytochemicals such as amino acids, 3-amino-2-naphthoic acid, cyclopentene-1-acetic acid, aminobenzoic acid, etc. present in banana pseudostem [19] have an influential role in plant growth, metabolism, and also anti-pathogenic activity on the plant [20–22]. In this regard, Mohapatra et al. [23] recorded high contents of K, P, Ca, and Mg in banana pseudostem sap. The soluble nutrients in any liquid fertilizer increase nutrient use efficiency through the minimize nutrient loss and ensuring better return [24, 25]. Similarly, organic liquid fertilizer-based nutrients and bioactive compounds improved growth, and yield, as well as created protection against disease and insect infestation of plants [26].

A previous study showed that the banana corm or hump extract increased the N, P, and K uptake of corn [21], and initiated the growth and yield of soybean [27]. The amendment of banana waste organic fertilizer increased the plant height, leaf size, and yield of cabbage [28] due abundance of inherent mineral nutrients. Similarly, N, P, K, and S foliar spray increase uptake of N and P as well as grain production [29]. Amal et al. [30] studied a foliar application of a mixed nutrient solution containing N, P, K, Mg, Zn, Fe, Mn, Cu, S, and B that had a significant effect on nutrient content in plants. Bio-stimulants in seaweed extract-initiated plant growth, biomass, and nitrogen assimilation in tobacco plants [31]. Foliar application of phosphorus (P) enhanced P use efficiency via uptake and translocation to the wheat grain [32]. The zinc concentration in maize, wheat, and rice grains increased by 63, 25, and 30%, respectively through the foliar spray of Zn over soil application [33, 34]. Likewise, iron deficiency results in reduced yields and nutritional quality of many crop plants [35]. The foliar use of amino acids had a positive effect on the growth, tuber yield, and nutrient content of cassava [36]. Moreover, foliar-applied plant extracts consistently increased nutrient uptake, growth, yield, and quality of sweet corn [37]. Previously, few studies evaluated the effects of banana stem compost on the yield of soybean [15] and sap on corn [38], fenugreek [39], and onion [40]. Besides, soil applications of inorganic fertilizers with banana pseudostem sap as foliar application resulted in influential effects on the pod yield of cowpea [41] in a neutral soil environment.

Nevertheless, the limited research on enriched banana pseudostem sap as a liquid organic fertilizer has been focused on and looks down upon the existence of micro nutrients in it to satisfy the yield potential and quality of corn. The corn production stability or enhancement, using minimum chemical fertilizers is the key challenge using the enriched banana pseudostem sap foliar application. The inadequate nutrients in fresh banana pseudostem sap as foliar fertilization or the sole application of chemical fertilizer may not meet-up the nutritional demand of crops. In this manner, the frequent spray with enriched banana pseudostem sap would be supplemented with soil-applied nutrients for better nutrient uptake, yield, and quality of corn. We hypothesized that the foliar application of enriched banana pseudostem sap with a lower fertilizer dose will reduce the chemical fertilizer use for corn production. To the best of our knowledge, this type of research is partial to crop production against acidic soil. Thus, the study has been executed to evaluate the effects of foliar application of enriched banana pseudostem sap with lower doses of chemical fertilizers on the growth, yield, and quality of sweet corn.

## 2. Materials and methods

### 2.1 Site description and soil

**Fig 1:**
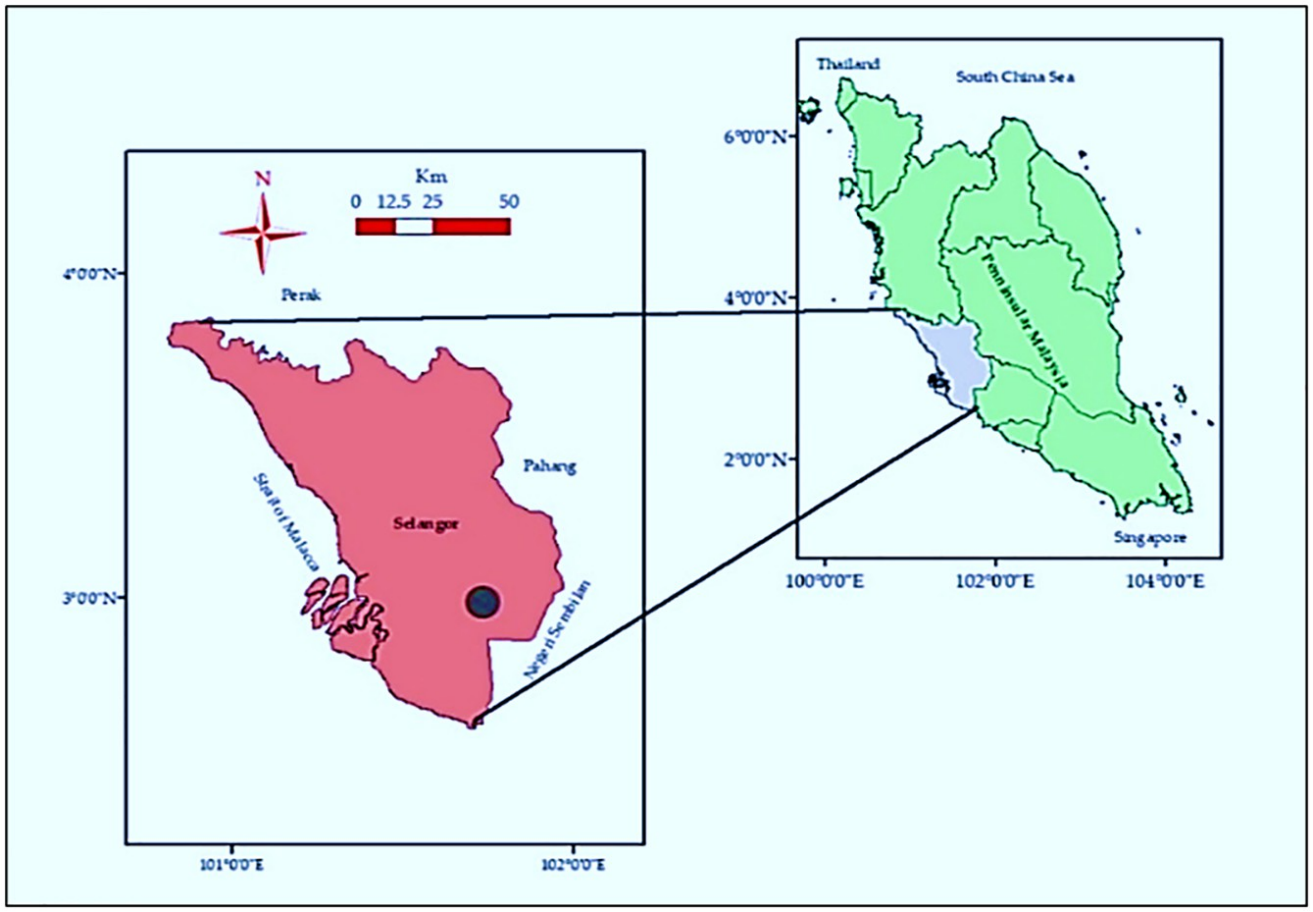
Geographical location of the study site (black circled).

**Fig 2:**
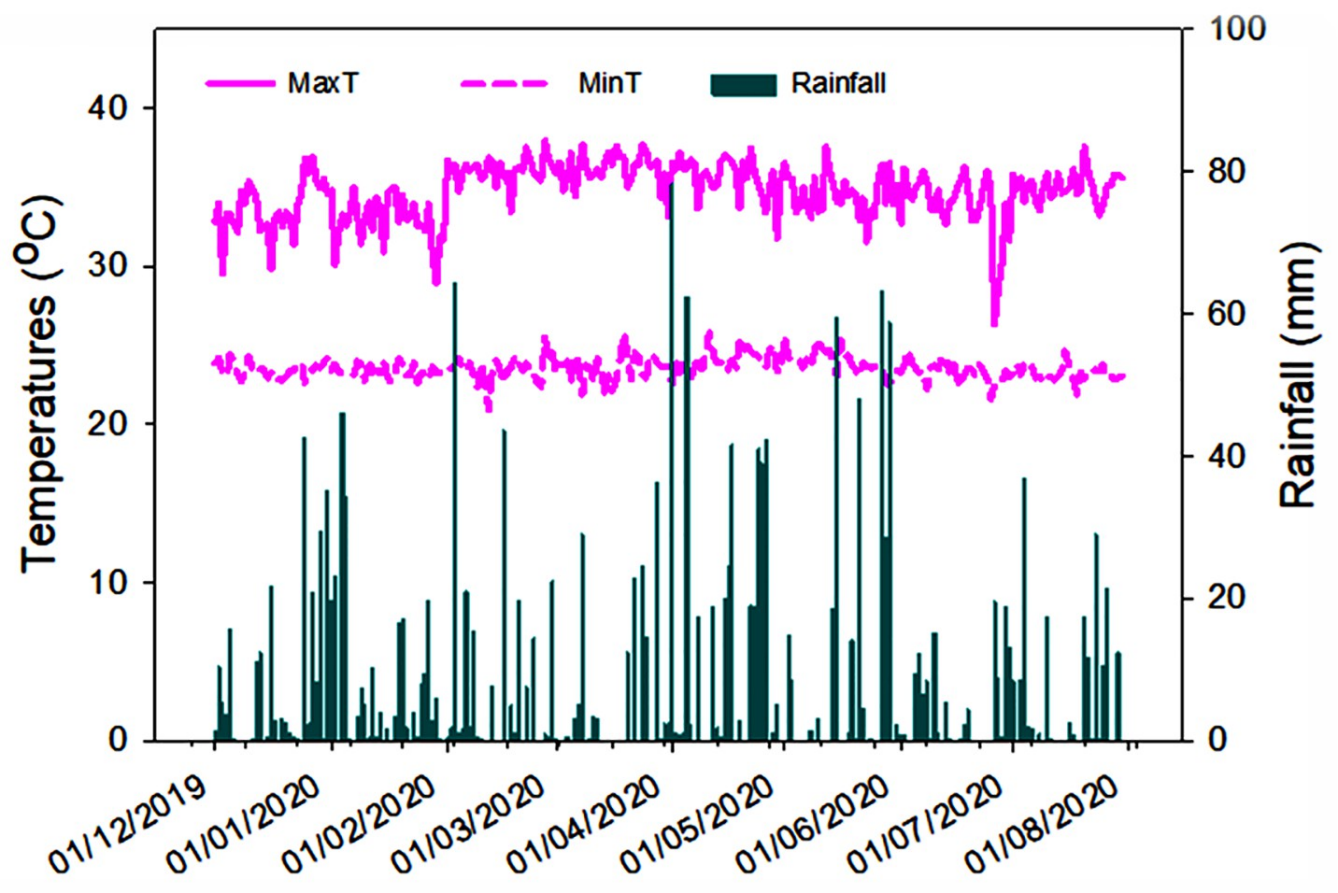
Temperatures and rainfall information during the field study period.

Two consecutive cycles of field studies were conducted in the same research field-15, under the Department of land management, Universiti Putra Malaysia from December 2019 to July 2020. The location of the field was latitude of 2°56′01.3″ N and longitude of 101°43′57.0″ E in the geographic position (Fig 1). The maximum temperature, minimum temperature and rainfall during the trial period were collected using LCD Digital Temperature and Humidity Meter (HTC-1, China) and shown in Fig 2. The soil was the order of Ultisol whose basic properties were tested before the initiation of the experiment and are presented in Table 1. The land was well-drained and slightly sloping and the soil was generally acidic, sandy-clay in texture, and low in available P.

**Table 1:** Initial soil chemical properties (0–25 cm depth) of the experimental field.

Parameters	Initial soil
pH	5.04±0.25
Organic matter (%)	1.12±0.08
Total Carbon (%)	1.20±0.05
Organic carbon (%)	1.09±0.02
Total N (%)	0.12±0.001
Carbon and nitrogen ratio	10±0.45
NH_4_-N (mg kg^-1^)	8.12±0.32
NO_3_-N (mg kg^-1^)	2.82±0.14
Cation exchange capacity (cmol_c_ kg^-1^)	3.94±0.13
Exchangeable K (cmol_c_ kg^-1^)	0.26±0.01
Available P (mg kg^-1^)	5.39±0.30
Exchangeable Ca (cmol_c_ kg^-1^)	0.91±0.07
Exchangeable Mg (cmol_c_ kg^-1^)	0.31±0.03

Value after ± indicates standard deviation which is the average of three replicated samples.

### 2.2 Collection and preparation of banana pseudostem sap

The banana pseudostem were collected from the banana field in Banting (2°48′17.1″ N and 101°30′12.99″ E), Selangor, Malaysia on 1st October 2019. The banana pseudostem were cleaned properly by washing them with fresh tap water. It was chopped into small sizes and separated from the leaf sheath. The leaf sheath was then passed through the electric crusher twice (locally made sugarcane crusher, ESM Machinery (JB) SDN, BHD, Malaysia) to collect the sap. About 60 liters of banana pseudostem sap was collected in a cleaned airtight bottle and preserved at 5 °C in the refrigerator for further use.

### 2.3 Enrichment of banana pseudostem sap

The banana pseudostem sap was enriched with N 20%, P_2_O_5_ 15%, K_2_O 15%, and boric acid 1.14% (20% N, 6.5% P, 12.45% K, and 0.2% B, respectively). In this regard, the used TSP fertilizer was finely grinded with an electrical grinder (Blue-Magic^®^, India). The enriched banana pseudostem sap was shaken for 6 hours using an orbital shaker at 160 rpm for proper mixing and then preserved at 5°C in the refrigerator.

### 2.4. Chemical analysis of both non-enriched and enriched banana pseudostem sap

The banana pseudostem sap (both the non-enriched and enriched) was shaken for 30 minutes by an orbital shaker at 160 rpm and directly measured the pH and EC followed by an automatic pH meter model HI 2211 (Hanna instrument, Woonsocket, Rhode Island, USA) and EC meter (Hanna 2300), respectively. The NH_4_-N and NO_3_-N were determined by using the distillation method [42]. The P, K, Ca, Mg, Na, Fe, Zn, Cu, Al, and B were determined from the non-enriched and enriched banana pseudostem sap separately by the Inductively Coupled Plasma (Perkin Elmer, Optima 8300) and spectroscopy (ICP) following 50 and 400 times dilution, respectively with distilled water [19]. The chemistry of banana pseudostem sap is shown in Table 2.

**Table 2:** Chemical properties of banana pseudostem sap.

Chemical characters	Banana pseudostem	
	Non-enriched sap	Enriched sap
pH	5.29±0.12	3.00±0.09
EC (μS cm^-1^)	6.47±0.20	-
Total N (g L^-1^)	4.25±0.15	200±9.21
Total P (g L^-1^)	0.92±0.02	66.31±4.17
Total K (g L^-1^)	1.92±0.04	123±5.40
Total Ca (mg L^-1^)	6.0±0.21	49.05±2.47
Total Mg (mg L^-1^)	83.39±3.80	83.39±4.78
Na (mg L^-1^)	2.30±0.08	2.30±0.11
Cu (mg L^-1^)	2.5±0.10	2.5±0.13
Zn (mg L^-1^)	1.0±0.03	1.0±0.05
B (g L^-1^)	0.25±0.01	3.25±0.13

Value after ± indicates standard deviation which is the average of three replicated samples.

### 2.5 Experimental design, treatments, and management

A consecutive field trial (two cycles) was conducted with three treatments (i) soil application of 100% chemical fertilizer (N_120_-P_60_-K_90_ kg ha^-1^) as control (T_1_) (ii) 75% soil-applied chemical fertilizers (N_90-_P_45-_K_68_ kg ha^-1^) with foliar application of non-enriched banana pseudostem sap (T_2_), (iii) 50% (N_60-_ P_30-_K_45_ kg ha^-1^) recommended chemical fertilizers with foliar application of enriched banana pseudostem sap were tested (T_3_) in the study to evaluate the banana pseudostem sap as organic liquid fertilizer alone or with chemical fertilizers on sweet corn planted in acidic soil. The study was designed in a randomized complete block design (RCBD) with four replications. The hybrid sweet corn Leckat 592 was tested at the plant-to-plant and row-to-row distance of 75 cm × 20 cm, and a unit plot size of 3 m × 2 m. Each unit plot had 40 plants. The nitrogen fertilizer was applied in two installments, 10 and 30 days after planting, while the full amount of P and K fertilizers were applied at the final land preparation. The seeds were sown followed by a 2-1-2 alternative pattern. The population density was maintained by gap-filling after three days of emergence. The non-enriched and enriched banana pseudostem sap were sprayed at the rate of 30 mL plant^-1^ with five installments at 15, 25, 35, 45, and 58, days after sowing. To prevent armyworm infestation, plants were sprayed with a mixture of banana pseudostem sap and pesticides from the first (15^th^ day) to the third (35^th^ day) installments. The last two installments (45^th^ and 58^th^ days) were sprayed with detergent @ 2g 10L^-1^ of the same spray solution to avoid the toxic effects of pesticide. Before spraying, 15% aqueous non-enriched sap and 1% enriched sap solution were ensured separately with distilled water. Irrigations were applied at each day interval considering the rainfall. The crop was harvested at the dough stage.

### 2.6 Data collection

#### 2.6.1 Measurement of SPAD value and photosynthesis in leaf

Chlorophyll content was measured at 45 days by using Soil Plant Analysis Development (SPAD) meter (SPAD 502 plus, Minolta, Japan) followed by Yuan et al. [43]. The portable LI-COR (LI-6400XT) was used to measure the rate of photosynthesis in the leaf at silking stage (45^th^ day).

#### 2.6.2 Growth and yield parameters

The data on agronomic parameters were the mean of two consecutive cycles of corn. The shoot length (cm) and cob length (cm) were measured by measuring tape while the root length (cm) was measured by measuring scale. The digital slide calipers were used to measure cob diameter (mm). The number of grain cob^-1^ was counted with the multiplication of the number of rows of grain in each cob and the number of grains in each row. In addition, the fresh cob yield (t ha^-1^) was measured from the whole unit plot (40 plants) by electric balance. Then the average weight of fresh cob per plant was multiplied by the number of plants in one hectare of land (66666) following the above-mentioned plant-to-plant and row-to-row spacing. The leaf area index was measured by using the equation [44].

$$\text{Leaf}\;\text{area}\;\left(\text{A}\right)=\text{L}\times \text{W}\times 0.75$$

Leafareaindex=(A×D×N)/10000 (1)

Where (A) express the area of a leaf (cm^2^), ‘L’ indicates the leaf length from leaf collar to leaf tip (cm), ‘W’ means the maximum width of the leaf (cm), the constant leaf area regression coefficient of 0.75 is related to leaf shape, the total leaf number of a sampled plant is N, plant density m^-2^ is D, and the factor 10,000 is used to convert from m^-2^ to cm^-2^.

The dry matter of the above-ground plant part and the cob was done by oven-dry method at 105°C for 24 hours and calculated with the equation;

$$\text{Dry}\;\text{matter}=\left({\text{W}}_{1}-{\text{W}}_{2}\right)\times 100/{\text{W}}_{1}$$ (2)

Where W_1_ is the initial weight and W_2_ means the weight after dry.

#### 2.6.3 Determination of nutrient concentration and uptake in plants and grain sample

The harvested cob and shoot samples were collected from the respective plot which was then cleaned and kept in paper bags, separately. The plant’s samples were analyzed from the second cycle crop to determine the nutrient concentration and quality parameters. The fresh cob samples were weighed in the paper bags, then dried in an oven at 70°C until a consistent weight. The dried materials were then ground and passed through a 4 mm sieve. The Leco TruMac CNS analyzer was used to calculate the total N. The total content of P, K, Ca, Mg, Cu, Zn, and Mn in plant and grain samples was extracted by the dry ashing method [45]. The amount of P was determined by auto-analyzer (Yellow method) from the same sample and the other elements was measured by atomic absorption spectrophotometer (A Analyst 800, PerkinElmer Corporation, Norwalk, Connecticut, USA). The nutrient uptake was estimated by multiplying with biomass (oven-dry weight) by nutrient concentration using the following equation [46];

$$\text{Nutrient}\;\text{uptake}=\frac{\text{Nutrient}\;\text{concentration}\;\left(\%\right)\times \text{dry}\;\text{biomass}\;\text{weight}\;(\text{g})}{100}$$ (3)

#### 2.6.5 Extraction and determination of total soluble protein

The soluble protein was extracted from corn samples following the Bradford assay method described by Ahmed et al. [47] and the extracted sample was read at 595 nm wavelength by UV-spectrophotometer (Model: Shimadzu. UV-160A Visible Recording Spectrophotometer, Japan). The formula used to calculate the soluble protein is;

$$\text{Total}\;\text{soluble}\;\text{protein}\;\left(\mu \text{g}\;{\text{g}}^{-1}\right)=(\text{C}\times \text{V})/(\text{W})$$ (4)

Here, C = Soluble protein (μg) content in the sample calculated from the standard curve line

V = Volume of the buffer solution used to extract the enzyme

W = Fresh weight of the sample taken for extraction

#### 2.6.6 Identification and quantification of amino acid

Acid hydrolysis of amino acid was performed by Cooper et al. [48] and then analyzed by High-Performance Liquid Chromatography (Waters, Alliance e2695). The total amino acid content was the sum of amino acid derivatives.

#### 2.6.7 Sample extraction procedure for determination of antioxidant

Samples extraction was done following the method described by [49] to measure the total phenolic content and antioxidant activity (DPPH Free Radical Scavenging Capacity). The absorbance was recorded at 765 nm and 517 nm wavelength, respectively by UV-spectrophotometer. The measurement was expressed as mg of gallic acid equivalent per 100 g of sample for phenolic content, while the percentage of antioxidant scavenging capacity was calculated by the equation as follows:

$$\text{\%}\;\text{DPPH}\;\text{scavenging}\;\text{capacity}=\frac{\text{Absorbance}\;\left(\text{blank}\right)\text{-Absorbance}\;(\text{sample})}{\text{Absorbance}\;(\text{blank})}\times 100$$ (5)

#### 2.6.8 Extraction and determination of sugar

Sugar profiling was done from the corn samples (dry) with some modification of Lu et al. [50] method and analyzed using high-performance liquid chromatography (HPLC) (Agilent, Santa Clara, California, USA). The total sugar content was the sum of sugar derivatives.

### 2.7 Benefit-cost analysis

Benefit-cost analysis was performed according to the method [51] where several equations were used as follows;

TotalGrossreturn=No.oftotalmarketablecob×Individualcobprice (6)

Totalcost=Totalvariablecost+Totalfixedcost (7)

Netreturn=Totalgrossreturn–Totalcostofproduction (8)

$$\text{Benefit-cost}\;\text{ratio}=\frac{\text{Total}\;\text{gross}\;\text{return}\;(\text{treatment})}{\text{Total}\;\text{cost}\;(\text{treatment})}$$ (9)

$$\text{Marginal}\;\text{benefit}\;\text{cost}\;\text{ratio}=\frac{\text{Net}\;\text{return}\;(\text{treatment})}{\text{Net}\;\text{return}\;(\text{control})}$$ (10)

### 2.8 Statistical analysis

ANOVA was done using PROC following RCBD design (SAS, 9.4). The combined analysis of the two consecutive trials’ data especially growth and yield found non-significant differences, therefore, the mean data of the two trials were presented in the results section. The nutrient content and qualitative data were considered for analysis from the second cycle of planted corn. The mean separation was compared by protected least significance differences (LSD) at a 5% probability level.

## 3. Results

### 3.1 Effects of banana pseudostem sap on physiological attributes of sweet corn

#### 3.1.1 SPAD value

**Fig 3:**
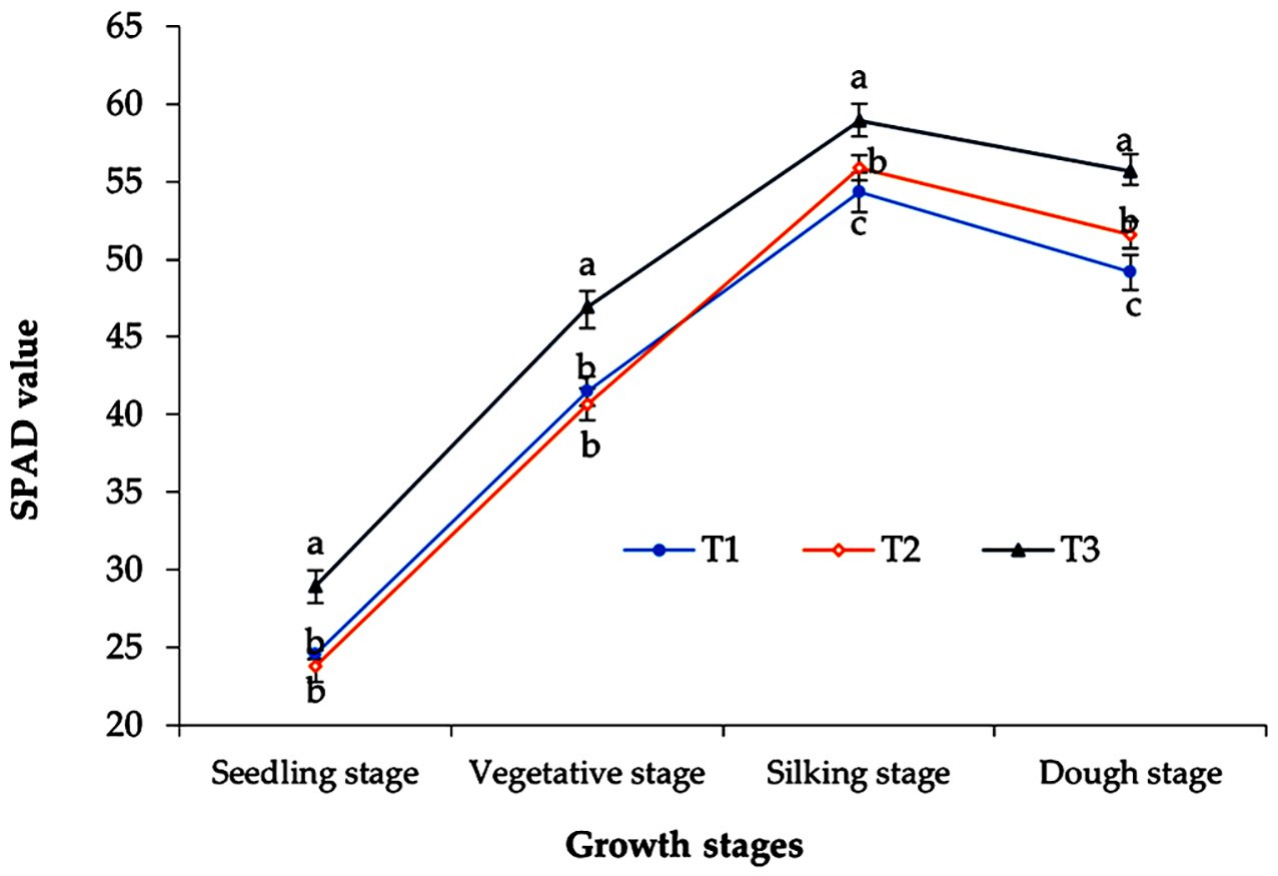
Effect of foliar spray of enriched banana pseudostem sap on the stage-wise chlorophyll content (SPAD value) of sweet corn. The vertical bars denote the standard error of means, and the value of each marker point indicates the mean of four replications (n = 4). The vertical bar denotes the ± standard error of mean the different letters are statistically significant with p≥ 0.05 of LSD test.

The SPAD value (greenness) showed an increasing trend up to the silking stage, after that, a little decline was observed in all treatments (Fig 3). The enriched sap (T_3_) treated plants provided a significantly higher SPAD value (28.9, 46.9, 59.0, and 55.7 in seedling, vegetative, silking, and dough stages, respectively) compared to sole chemical fertilizers (23.8, 40.6, 54.3, and 49.2 in seedling, vegetative, silking and dough stage, respectively) (T_1_). The chlorophyll content in T_2_ was very close to the T_1_ treatment in the study periods while the silking stages showed greater chlorophyll content in all treatments than in other crop growth stages.

#### 3.1.2 Photosynthesis

**Fig 4:**
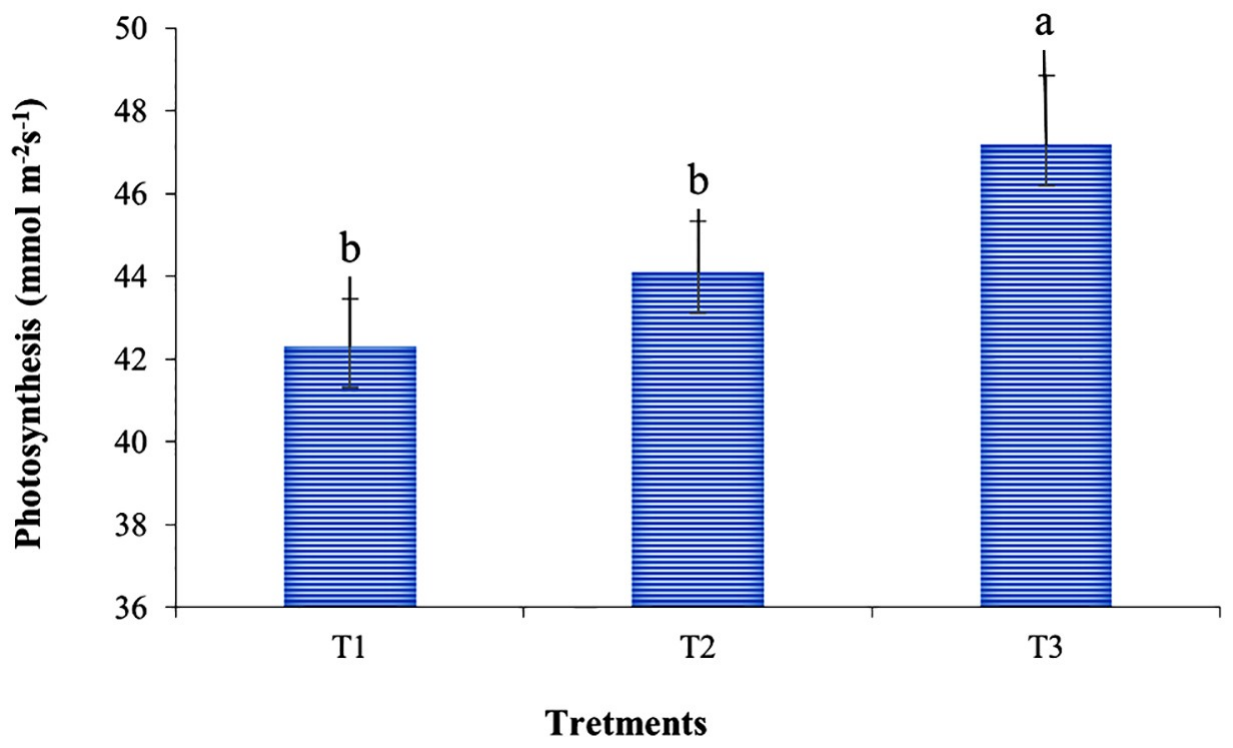
Effects of enriched banana pseudostem sap on the photosynthesis rate of corn plants. The vertical bar denotes the ± standard error of mean the different letters are statistically significant with p≥ 0.05 of LSD test.

The photosynthesis rate of corn plants was significantly affected by the foliar application of enriched banana pseudostem sap (Fig 4). The photosynthesis rate was found to be higher (47.2 mmol m^-2^ S^-1^) in enriched sap (T_3_) treated plants compared to T_1_ (42.3 mmol m^-2^ S^-1^) and T_2_ (44.1 mmol m^-2^ S^-1^) treatments. Although the foliar spray of non-enriched sap (T_2_) showed a statistically identical but numerically higher value to the T_1_ treatment. The foliar application of enriched banana pseudostem sap (T_3_) increased photosynthesis by 12% while the non-enriched sap (T_2_) increased by 4% compared to soil-applied chemical fertilizers.

### 3.2. Effects of enriched banana pseudostem sap on grain nutrient content of sweet corn

The nutrient concentration in grain was varied due to the combined use of soil-applied chemical fertilizers with foliar fertilization of banana pseudostem sap (Table 3).

**Table 3:** Effects of banana pseudostem sap on the grain nutrients content.

Parameters	Treatments			LSD (0.05)
	T_1_	T_2_	T_3_	
Nitrogen (g 100 g^-1^)	1.74b	1.77b	1.93a	0.09
Phosphorus (g 100 g^-1^)	0.21a	0.20a	0.20a	0.02
Potassium (mg 100 g^-1^)	836b	874b	932a	54.4
Calcium (mg 100g^-1^)	53.0b	53.37b	66.58a	5.27
Magnesium (mg 100 g^-1^)	5.69c	7.05a	6.62b	0.38
Zinc (mg 100 g^-1^)	20.98b	25.63a	21.7b	0.87

The mean value of four replicated samples in the row as the different letters are statistically significant with p≥ 0.05 of LSD test.

The highest content of N (1.93 g 100 g^-1^) was measured from T_3_ compared to T_1_ (1.74 g 100 g^-1^) and T_2_ (1.77 g 100 g^-1^) treatments. A similar trend was observed in the case of grain K and Ca concentrations of corn. The Mg and Zn concentration varied significantly by the foliar application of banana pseudostem sap. Results showed that the highest concentrations were 7.05 and 25.6 mg 100 g^-1^ of Mg and Zn, respectively which was recorded in T_2_ treatment while the lowest Mg (5.69 mg 100 g^-1^) and Zn (21.0 mg 100 g^-1^) were recorded in T_1_ treatment. It is calculated that the grain nutrients concentration of N, K, Ca, Mg, and Zn, in T_3_ was increased by 10.9, 11.5, 25.6, 16.3, and 3.48%, respectively over the T_1_ treatment.

### 3.3 Nutrient uptake of sweet corn

**Fig 5:**
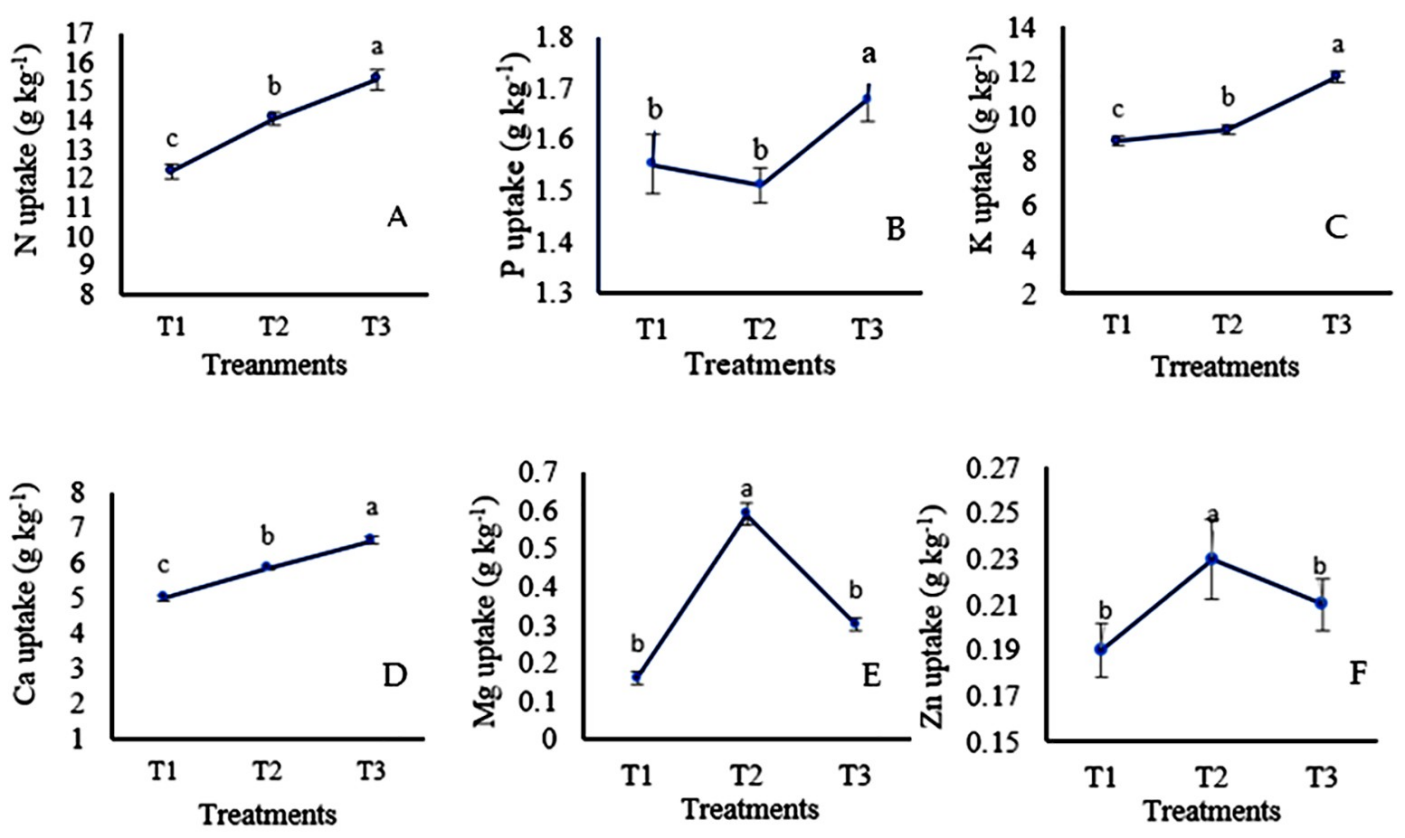
(A-F). Effects of enriched banana pseudostem extract on total nutrient uptake of sweet corn. The vertical bar denotes the ± standard error of mean the different letters are statistically significant with p≥ 0.05 of LSD test.

The nutrient uptake by sweet corn was varied due to the foliar application of banana pseudostem sap (Fig 5A–5F). The result showed that the dual application of soil-applied chemical fertilizers with foliar spray of enriched banana pseudostem sap has significantly increased the nutrient uptake by corn compared to the control. Of the three treatments, T_3_ showed superior results in terms of nutrient uptake except for Mg and Zn. From the results, it is apparent that 15.40, 1.68, 11.74, 6.64, 0.30, and 0.210 g kg^-1^ of N, P, K, Ca, Mg, and Zn, respectively in T_3_ treatment which increased by 25.81% N, 8.38% P, 32.80% K, 32.53% Ca, 87.5% Mg, and 10.52% Zn, over the control (T_1_). The results also demonstrated that N, phosphorus (P), potassium (K), calcium (Ca), magnesium (Mg), and Zinc (Zn), were increased by 14.8, -2.58, 5.56, 16.6, 250, and 21.05% in T_2_ treatment over the control, respectively. However, the magnesium and zinc uptakes in the plant were significantly higher in T_2_ followed by T_1_ and T_3_ treatments.

### 3.4 Growth and yield components of sweet corn

The root length, leaf area index, the number of grains, fresh cob yield, and biomass weight of corn was varied by the fertilizer application (Table 4). The treatment of 50% soil-applied chemical fertilizers with foliar spray of enriched banana pseudostem sap (T_3_) resulted in superior growth and yield performance than that of 75% soil-applied chemical fertilizers with non-enriched (T_2_) sap as foliar and sole chemical fertilizers (T_1_) of soil application. In this study, combined fertilization in soil and foliar spray of enriched sap increased root length, leaf area index, number of grains, cob yield, and dry biomass by 12, 11.3, 35.0, 39.0, and 29.0%, respectively over control. The results of the T_1_ and T_2_ treatments were statistically identical while T_2_ performed numerically better results than the T_1_ treatment.

**Table 4:** Effects of enriched banana pseudostem sap on the growth and yield of sweet corn.

Treatments	Root length (cm)	Leaf area index	Grains cob^-1^ (no.)	Fresh cob yield (t ha^-1^)	Dry biomass yield (t ha^-1^)
T_1_	57.1b	3.02b	371b	13.74b	6.84b
T_2_	60.3ab	3.08b	405b	15.59b	7.12b
T_3_	64.3a	3.36a	501a	19.16a	8.83a
LSD (0.05)	5.24	0.14	66.28	3.77	0.88
Sig. level	*	*	**	*	**

The mean values in the column as the different letters are statistically significant with p≥ 0.05 of LSD test.

### 3.5 Enriched banana pseudostem sap on the nutritional quality of sweet corn

#### 3.5.1 Protein and amino acid composition

**Fig 6:**
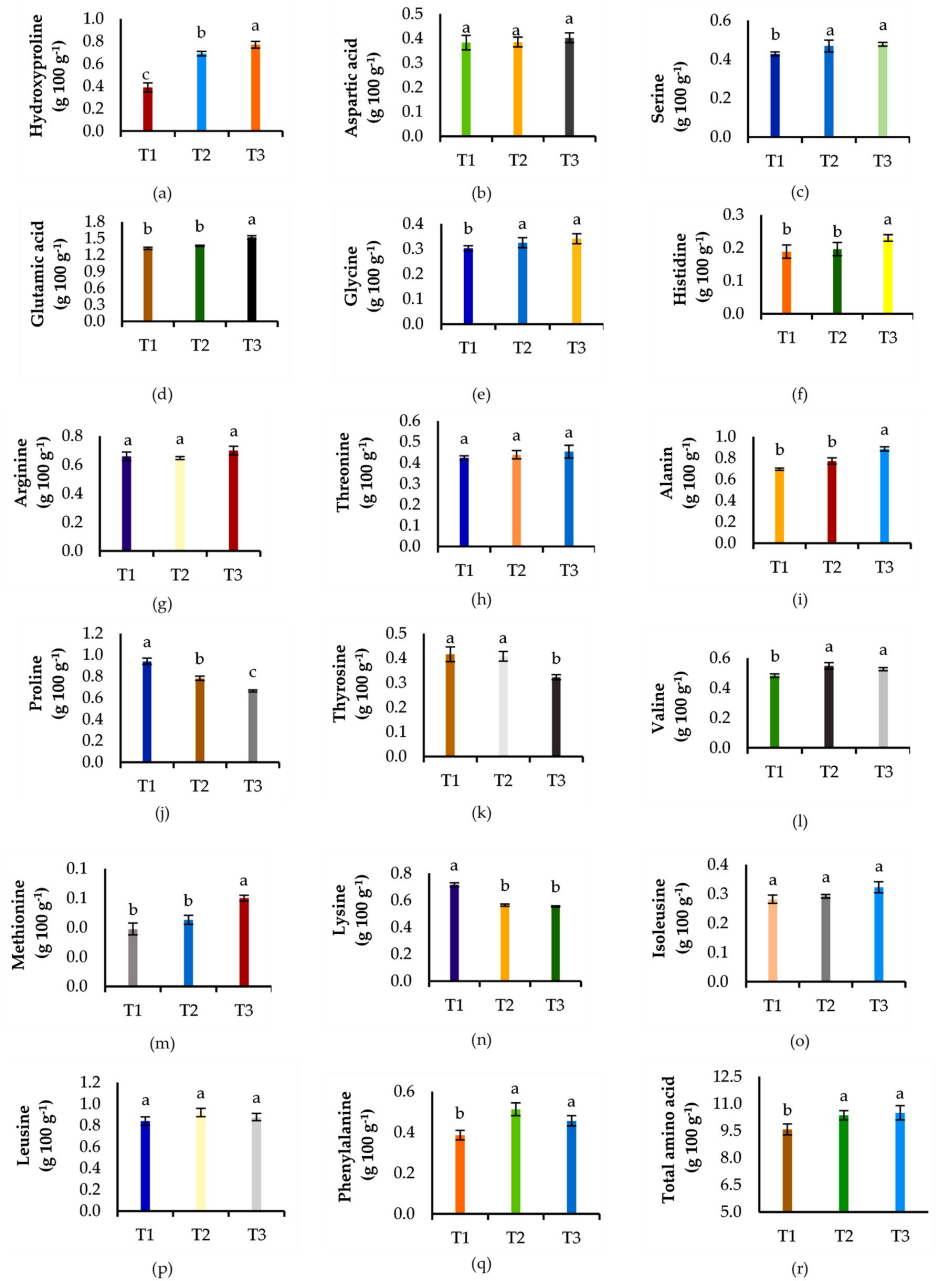
**(a—r)**. Combined application of soil-applied fertilizers with foliar spray of banana pseudostem sap influenced the amino acids composition as well as total amino acids content. Mean ± standard error within the bar followed by the different letters are significantly different at p ≥0.05 (LSD test).

The protein and amino acid composition of sweet corn was varied due to the foliar application of banana pseudostem sap. The highest protein content (24.5 g 100 g^-1^) was obtained from foliar application of enriched banana pseudostem sap, followed by non-enriched sap (22.1 g 100 g^-1^) while the lowest protein and amino acid (21.0 g 100 g^-1^) were found in soil-applied chemical fertilizers (Fig 6a–6q).

In this case, the total soluble protein showed a significant (p≤0.05) increase by 5.23% and 16.5% in T_2_ and T_3_ treatments, respectively compare to soil-applied chemical fertilizers (T_1_). Results represented that both the non-enriched or enriched banana pseudostem sap treated plots provided significantly (p≤0.05) higher concentrations of most of the amino acids (Fig 6a–6q). Among the amino acids (17), aspartic acid, arginine, threonine, isoleusine, and leusine had no remarkable variations by foliar fertilization (Fig 6).

But the significantly higher content of hydroxyproline, serine, glutamic acid, glycine, histidine, alanine, valine, methionine, and phenylalanine was measured in the foliar spray treatments, especially in enriched banana pseudostem sap over the soil-applied chemical fertilizers which fairly reflected on total amino acid content. In this regard, the highest (10.5 g 100 g^-1^) total amino acid was measured in enriched sap (T_3_) followed by (10.36 g 100 g^-1^) non-enriched banana pseudostem sap (T_2_) compared to (9.58 g 100 g^-1^) in chemical fertilizers plots (T_1_). The total amino acid concentration over soil-applied chemical fertilizers was increased by 9.60% due to foliar application of enriched banana pseudostem sap (Fig 6a–6r).

#### 3.5.2 Phenolic content, DPPH scavenging capacity and sugar content of corn

The quality of corn was significantly influenced by the foliar application of banana pseudostem sap as shown in Table 5. The DPPH scavenging capacity and total phenolic content showed an increasing trend that was 4.43 to 7.38% and 3 to 27.8% in T_2_ and T_3_ treatments, respectively over the sole chemical fertilizers due to the free radicle scavenging capacity of phenolic compounds. The results revealed that as compared to soil-applied chemical fertilizers, the sugar content was considerably higher by 4.26% and 15.5% from T_2_ and T_3_ treatments, respectively. In this study, applied banana pseudostem sap contained a considerable amount of macro and micronutrients, especially K, Ca, and Mg that could gain in increasing sugar content than that of soil-applied chemical fertilizers.

**Table 5:** Effects of foliar application of enriched banana pseudostem sap on different quality parameters of sweet corn.

Parameters	Treatments			LSD (0.05)
	T_1_	T_2_	T_3_	
Total soluble protein (g 100 g^-1^ DW)	21.0b	22.1ab	24.5a	2.58
Phenolic content (mg gallic acid g^-1^ FW)	0.36b	0.37b	0.46a	0.04
DPPH Scavenging capacity **(%)**	74.0b	77.3a	79.5a	2.23
Sucrose (g 100 g^-1^ DW)	2.80a	2.88a	3.04a	0.60
Glucose (g 100 g^-1^ DW)	0.80b	0.83b	1.05a	0.14
Fructose (g 100 g^-1^ DW)	0.86b	0.87b	1.06a	0.11
Total sugar (g 100 g^-1^ DW)	4.46b	4.65b	5.15a	0.43

The means value of four replicated samples within the row followed by the different letters is significantly different at p ≥0.05 (LSD test). FW, fresh weight; DW, dry weight

#### 3.5.3 Relationship among the nutrient uptake, growth, yield and quality factors of corn

**Fig 7:**
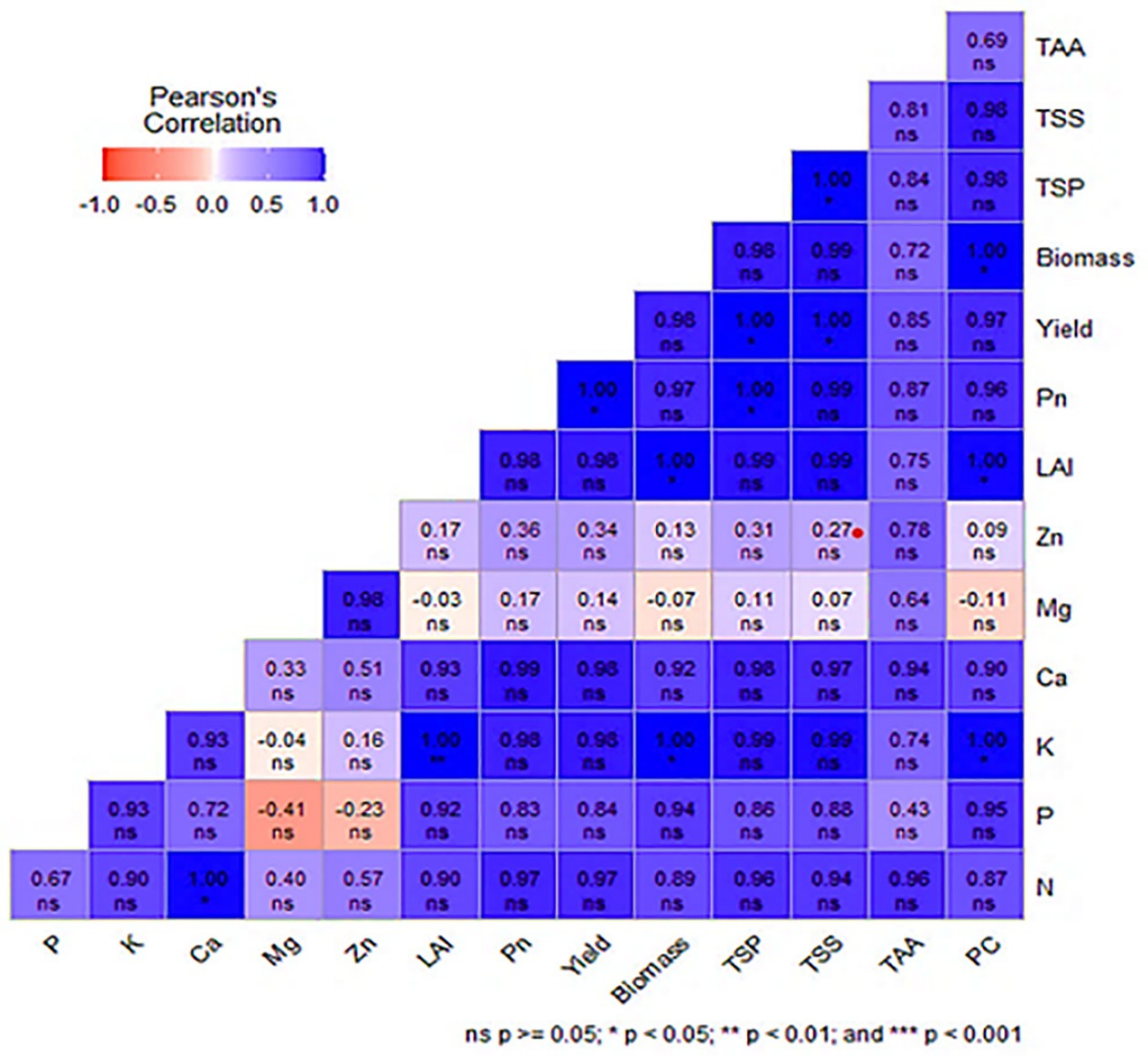
Correlation among the nutrient uptake, growth, yield and quality of corn. LAI: Leaf area index, Pn: Photosynthesis rate, TSP: Total soluble protein, TSS: Total sugar content, TAA: Total amino acid and PC: Phenolic content.

Pearson’s correlation analysis showed potassium uptake had strong and significant relationship with leaf area index, biomass and phenolic content while the cob yield had a positive and significant correlation with Pn, TSS and TSP (Fig 7). Besides, the negative and non-significant relationship was found from P with Mg and Zn uptake, and K with Mg uptake. Similarly, Mg showed weak and negetive relationship on leaf area index, biomass and phenolic content. These relationship mean that the K uptake from enriched banana pseudostem sap influenced growth, yield and quality of corn.

#### 3.5.4 Economic analysis

The highest total return (RM. 51496) was found in the combined application of soil-applied fertilizers with foliar spray of enriched banana pseudostem sap (T_3_) treatment while the lowest return (RM. 44840) was in the sole chemical fertilizers (T_1_) (Table 6). Considering the total cost of production, the lowest cost involvement was found from the treatment with foliar spray of enriched banana pseudostem sap (Table 7). Similarly, the superior benefit-cost ratio (4.14) and marginal benefit-cost ratio (1.29) were also higher for treatment T_3_ and the lowest for treatment T_1_ (Table 7).

**Table 6:** Effects of foliar application of enriched banana pseudostem sap on value addition of corn.

Treatment	Marketable cob (no.)	Non-marketable Cob (no.)	Large size (no.)	Price cob^-1^ (RM)	Total price (RM)	Medium size (no.)	Price cob^-1^ (RM)	Total price (RM)	Small size (no.)	Price cob^-1^ (RM)	Total price (RM)	Average price cob^-1^ (RM)	Total return (RM)
T_1_	60800	3200	21280	1.0	21280	27360	0.75	20520	12160	0.25	3040	0.74	44840
T_2_	62080	1920	24211		24211	30772		23079	7440		1862	0.85	49152
T_3_	62080	1920	27632		43456	30144		22608	5024		1256	0.87	51496

The total return is the sum of the total price of different sizes of corn, large size ≥15 cm (35%), medium size ≥10 to 14 cm (45%), small size ≤10 cm (20%) for T_1_ and large size ≥15 cm (39%), medium size ≥10 to 14 cm (49%), small size ≤10 cm (12%) for T_2_ while large size ≥15 cm (44%), medium size ≥10 to 14 cm (48%), small size ≤10 cm (8%) for T_3_ treatment.

**Table 7:** Effect of foliar application of enriched banana pseudostem sap on the economic performance of corn.

Treatments	Total return (RM)	Total cost (RM)	Net return (RM)	Benefit-cost Ratio (BCR)	Marginal benefit cost ratio (MBCR)
T_1_	44840	14682	30158	3.05	-
T_2_	49152	14547	34605	3.38	1.15
T_3_	51496	12421	39075	3.74	1.29

Non-material cost; Land hire and preparation (600+1000 RM), labour hire cost; (72* × 65 RM) = 4680 RM, grading, marketing; 2000 RM and material cost; 6102 RM (seed, fertilizers, insecticides, and irrigation) and miscellaneous; 800 RM (plastic canvas, gunny bags, twist, etc.) were considered for treatment T_1_, while T_2_ and T_3_ treatments added 75% and 50% chemical fertilizers cost, respectively with banana pseudostem sap preparation and enrichment cost; 15 RM + (6* × 65 RM) = 405 RM. All other costing was the same as T_1_ treatment. * mean no. of labour used.

## 4. Discussion

### 4.1 Chlorophyll content

The enriched banana pseudostem sap significantly improved the SPAD value of corn at all growth stages might be due to the abundance of macro and micronutrients (N, P, K, Ca, Mg, and Zn) in the foliar application of enriched sap (T_3_) and their faster absorption helped increase the chlorophyll synthesis significantly. According to Duarte et al. [52], the repeated spraying within the vegetative-silking stage ensured better nutrient uptake by corn might be the cause of higher chlorophyll content. Besides, banana pseudostem contains bio-active molecules such as amino acids [53], proline, betaine, and glutamic acid that have stimulatory effects on chlorophyll synthesis [54] which could be promoted a higher SPAD value in T_3_ treatment. In the senescence stage, chlorophyll catabolites naturally broke leading to the degradation of SPAD value in the dough stage [55]. That’s why all the treatments showed a declining chlorophyll content after the silking stage (Fig 3).

### 4.2 Photosynthesis

Foliar application of enriched banana pseudostem sap with soil-applied chemical fertilizer (T_3_) significantly increased the photosynthesis rate of corn (Fig 4) might be due to an improved level of inherent and enriched K in banana pseudostem sap (Table 2). This agreement was supported by the findings of Ali et al. [56], who reported that foliar spray of K_2_O solution increased the photosynthesis by 61.6% of corn over the control. Besides, the ionic forms of nutrients in enriched banana pseudostem sap being absorbed with water have increased the leaf area which thereby may help to capture more light for higher photosynthesis [57]. Moreover, the uptake of N, P, K, Ca, and Mg by plants have an influential role in electron transport and photosynthetic activities [58] and these nutrients were available in banana pseudostem sap (Table 1). Besides, the appropriate content of P, K, Ca, or Mg strongly influenced plant metabolism [59], which was responsible for higher photosynthesis [56].

### 4.3 Nutrient uptake by corn

The combined effects of soil-applied fertilizers with foliar-applied banana pseudostem sap fairly influenced the nutrient uptakes of corn (Fig 5A–5F). In the study, soil-applied fertilizers with foliar spray of enriched sap (T_3_) provided higher nutrient uptake except for Mg and Zn. This might be the fact of opening the stomata by leaf hydration [60] that allowed the rapid access of foliar spray into the leaves [5]. Results were also consistent with the findings of Pangaribuan et al. [37] who explored the higher uptake of N, P, and K in sweet corn from the combined application of soil-applied fertilizers and foliar spray of plant extract. Likely, the N, P, and K content of corn were found 38.75, 18.16, and 16.92% higher by the foliar application of amino acids containing organic liquid fertilizer reported by Tadros et al. [61]. Similarly, the higher N, P, K, and S concentrations in soybean plants have been found from foliar spray with 15% aqueous seaweed extract [62]. In this study, non-enriched banana pseudostem sap (T_2_) resulted in higher Mg and Zn uptake than T_3_, which might be due to the antagonistic effect of high concentrations of K and Ca with the Mg and Zn in the enriched banana pseudostem sap (Table 3) as supported by Sun et al. [63]. Moreover, the presence of glutamic acid, proline, and benzoic acid in banana pseudostem sap [53] could have induced better root growth which might be helped the higher nutrient uptake by plants [64, 65]. In the control treatment (T_1_), nutrient uptake showed a significantly lower value due to the fixation or leaching loss caused by soil-nutrient interactions in low-pH soil [5]. Therefore, soil-applied chemical fertilizers with foliar spray of enriched sap (T_3_) resulted in higher nutrient uptake compared to the control (T_1_) treatment.

### 4.4 Yield and yield contributing characters of corn

In this study, soil-applied chemical fertilizers with enriched banana pseudostem sap promoted 11.25, 39, and 29% higher leaf area index, fresh cob yield, and biomass, respectively over the soil-applied chemical fertilizers (Table 4). These findings were corroborated by various liquid organic fertilizers studies. For example, the foliar application of amino acids containing liquid organic fertilizer was increased by 6.6–23% LAI and 17–32% higher maize yield [24]. Likely, Aulya et al. [66] observed a significant increase in maize leaf area through the application of 10% organic extract. Cassim et al. [67] studied that foliar application enriched N solution increased by 14.7% higher corn yield over the control. Similarly, Paramesh et al. [68] obtained higher cob yield and biomass of wheat compared to 100% RD of soil application. The cause of our findings may be due to the faster absorption of nutrients in the sprayed sap by the leaves without interfering with any soil-related limitations [30]. Moreover, the secondary metabolites in banana pseudostem sap, such as 4-aminobenzoic acid, glutamic acid, cyclopentene-1- acetic acid, proline betaine, butanoic acid, hydroxysouric acid, and aluminium acetate related to promoting plant growth and development [53]. Application of enriched sap as foliar spray through frequent splitting supported to meet-up the nutrients demand which helped to escape the soil-related constraints. In the current study, enriched N in banana pseudostem sap was absorbed quickly, and increased leaf area, photosynthesis, and shoot height which contributed to higher biomass [69]. This means that frequent application of enriched sap optimized both macro and micronutrients, which in turn, enhanced the growth and yield attributes of corn in acidic soil.

### 4.5 Nutritional quality of corn

The protein, amino acids, phenolic contents, and sugar contents are the major indicator of the quality of sweet corn which is governed by the availability of nutrients either from soil-applied fertilizers and/or foliar fertilization. In our study, the foliar-applied banana pseudostem sap was rich in K, Ca, and Mg as well as contain some extent of micronutrients (Table 2) that was in agreement with Islam et al. [14]. Moreover, inherent nutrients with enriched N, P, K, and B in the banana pseudostem sap created an enriched liquid fertilizer (Table 2). Thus, the foliar spray of enriched sap provided 16.5% higher protein content of corn which was consistent with the finding of Ali et al. [56] who recorded 15.3% higher protein in maize over control. This might be possible from the balance of macro and micro-nutrients and their faster absorption by the shoot. Besides, the soil-applied P and enriched P in the banana pseudostem sap have been found to enhance better root growth, N uptake, and metabolism, thereby the protein concentration in grain increased considerably [70]. Similarly, acid soil had limited P availability leading to a reduction of nitrogen acquisition in the plant, thereby reducing protein and amino acid synthesis which occurred in sole chemical treatment (T_1_). In the same study, Gao et al. [70] showed a positive correlation of zinc with protein and amino acid in wheat. Likely, Zn in banana pseudostem sap may have stimulated the function of indole acetic acid thereby contributing to higher protein and amino acid content [68]. The soluble nutrients in banana pseudostem sap have been gained by plants. In this regard, P excited the nitrate reductase to efflux nitrate in the internal pathway and later on stored in the embryo which is the key component of protein and amino acid [71]. In terms of leaf hydration, rapid access to ions, and nutritional balance in a plant, K might be activated by enzymes and photosynthesis which could increase sugar content, free amino acids, and anti-oxidant content (Table 5). The findings were supported by Bakry et al. [72] who studied the banana peel extract with tryptophan on the quinoa plant. Thus, the enriched banana pseudostem sap could be a reliable nutrient supplementing organic liquid fertilizer to improve the quality of corn.

### 4.6 Economic performance of corn

Cost-benefit analysis is an important tool to test the economic viability of an approach or product which includes the relevant materials and non-material costs that fairly influenced the net income [51]. In the current study, the high price of a maximum number of improved-size corn (Table 7) in T_3_ treatments contributed to the increased value of total income (RM. 51496). Besides, the same treatment required half (50%) of the chemical fertilizers applied to T_1_ decreasing the total cost, ultimately resulting in a net higher income (RM. 39075), benefit-cost ratio (4.14), and marginal benefit-cost (1.29) (Table 7).

## 5. Conclusion

The application of enriched banana pseudostem sap as foliar spray with 50% recommended dose of soil-applied fertilizer increased fresh cob yield by 39% compared with the 100% chemical fertilizer. The combined effects of soil-applied fertilizers with enriched banana pseudostem sap also promoted the protein content (16.5%), amino acid (9.6%), phenolic content (27.8%), antioxidant capacity (7.4%), and sugar content (15.5%) of corn compared to the sole chemical fertilizer. Besides, the higher BCR and MBCR values confirmed that the foliar application of enriched banana pseudostem sap is a profitable option. Thus, foliar-sprayed enriched banana pseudostem sap can be utilized as a supplementary nutrient source or organic liquid fertilizer but it is recommended that more validation of the findings from the multilocation trials is needed in acid soil.
